# Lipid patterns, alcohol intake and BMI of adult Nigerians in a sub-urban slum in Enugu, Nigeria

**DOI:** 10.11604/pamj.2014.18.37.1926

**Published:** 2014-05-10

**Authors:** Gladys Ifesinachi Ahaneku, Joseph Eberendu Ahaneku, Charles Ukachukwu Osuji, Celestine Ogonna Oguejiofor, Patience Chioma Opara

**Affiliations:** 1 Department of Medicine Nnamdi Azikiwe University and Teaching Hospital, Nnewi Campus, Nigeria; 2 Chemical Pathology, Nnamdi Azikiwe University and Teaching Hospital, Nnewi Campus, Nigeria

**Keywords:** Lipid patterns, Nigerians, alcohol intake, Body mass index (BMI), slum

## Abstract

**Introduction:**

Demonstration of cardiovascular disease (CVD) markers in healthy subjects with normal blood chemistry tests underscores the need to study social determinants of risk factors to aid primary prevention worldwide; particularly in slums which harbor nearly 80% of rural to urban migrants in the epidemiologically transiting Africa where CVDs were previously unknown. The objective of this study was to assess lipids in relationship to alcohol consumption and BMI in a Nigerian slum.

**Methods:**

Cross sectional community based prevalent study involving 191 apparently healthy inhabitants aged 18- 85 years recruited by convenient sampling. Heights, weights and BMIs were measured/ calculated, venous blood samples collected and lipid analysis done procedurally. Excel 13 and SPSS statistical soft ware were used for analysis and chart representation.

**Results:**

Their mean parameters were: Age (43.87 ± 1.62 years), triglycerides (TG; 1.20 ± 0.08mmol/L), total Cholesterol (TC; 4.54 ± 1.70mmol/L), low density lipoprotein cholesterol (LDLC; 3.69 ± 1.69mmol/L), high density lipoprotein cholesterol (HDLC; 0.61 ± 0.24mmol/L), RPI (7.12 ± 5.24), body mass index (BMI; 25.08 ± 5.18Kg/M2). TG and HDLC values were lowest in obese non alcohol drinkers while all other lipid parameters increased with BMI in both drinkers and non drinkers. Low HDLC prevalence was lowest in obese alcohol drinkers and highest (100%) in their non drinking counterparts. Having favourable HDLC was highest in daily alcohol consumers. No weekly drinker (0%) had favourable HDLC.

**Conclusion:**

Ignorance, poor nutritional and health education may be major factors in the strategic challenge posed by the emergence of non communicable diseases in Africans.

## Introduction

With inappropriate socio-economic status determination (income no longer tallies with level of academic attainment), rapid urbanization, sedentary lifestyle and westernization of diets; which are the bane of epidemiological transition in Africa, morbidity and mortality from coronary vascular diseases (CVD) is posing a strategic challenge to the health of the average African [1].

Misappropriate economic and socio-cultural values, poor nutritional education, physical inactivity, use of automobile and ignorance all seem to be contributory to the emergence of hypercholesterolemia and other cardiovascular risk factors in African communities where these were previously unknown.

Recent publications [2] underscore the importance of strategies that aim to improve prevention in people without existing cardiovascular disease (primary prevention) in managing the overall burden of CVD worldwide, hence, the need to study the social determinants of cardiovascular risk factors [3] in different parts of the world cannot be over emphasized. Nigeria; the most populous African nation, most likely stands to bear a heavy bulk of the impact of the emergence of cardiovascular risk factors in the rapidly epidemiologically transiting Africa. Therefore, to assess the health impact of epidemiologic transition in Africa, Nigeria is a nation to consider and since over 43% to 78.2% percent of people migrating from rural areas to urban cities in Africa end up in slums [4], a target of this population is of utmost importance in defining and tackling the emerging health problems of Africa at the grass root/ primary health care level. This study, therefore, assessed lipids and its relationship with alcohol intake and body mass index (BMI) in a “non commercial” suburban slum in South East Nigeria.

## Methods

This study was a cross sectional community based prevalence study. Ethical approval and individual consent were appropriately obtained before the study was embarked on or participants recruited. Six medical officers assisted in the study. Each person's height and weight were taken procedurally, BMI calculated and a questionnaire incorporating relevant data and alcohol history administered. Venous blood sample was also into a clean plain bottle for lipid analysis, later done in the chemical pathology laboratory of NAUTH, Nnewi. All participants who indicated interest in having their lipid profile results sent to them were duly obliged after the lipid assay.

**Data analysis:** SPSS (16.0) statistical soft ware was used for statistical analysis.

## Results

Participants in this study were 191 (mean age: 43.87 ± 1.62 years). In the general population as well as in those that consumed alcohol, the mean values of all their lipid parameters in increased with their BMI except for HDLC (Table 1). All forms of dyslipidemias increased as obesity tendency and overt obesity increased in alcohol consumers but in non-alcohol consumers, high TG was highest in those with above normal BMI (Table 2). Tendency to have high (favorable) HDLC was highest in daily alcohol consumers (8.4%) and none (0%) in weekly consumers. Tendency to have favorable HDLC was highest in those with higher than normal BMI.

**Table 1:** Mean parameter of Participants in relation to alcohol Consumption and BMI

		Alcohol drinkers				Non-alcohol drinkers			
	Normal Range	Those with Normal BMI	All those with BMI ≥ 25Kg/M^2^	Only those Overtly Obese	All Alcohol drinkers	Normal BMI	All those with BMI ≥ 25Kg/M^2^	Overtly Obese	All Non-alcohol drinkers
**Age**	≥ 18	46.29±1.65 n = 38	47.16± 1.24 n = 29	41.42± 1.06 n = 12	46.69± 1.47 n = 70	41.61± 1.84 n = 71	43.65± 1.42 n = 48	42.08±9.06 n = 12	42.43±1.68 n = 110
**TG**	0.5-.71	1.24± 0.94 n = 37	1.25± 0.93 n = 29	1.54 ±1.18 n = 11	1.24± 0.93 n = 66	1.07± 0.78 n = 63	1.35± 1.84 n = 46	1.22 ± 0.79 n = 12	1.19± 0.81 n = 110
**TC**	3.6-.17	4.17±1.68 n = 37	4.52±1.50 n = 29	4.85± 0.93 n = 11	4.32± 1.60 n = 66	4.50± 1.52 n = 63	4.90± 2.05 n = 46	5.54± 1.73 n = 12	4.67± 1.76 n = 110
**LDLC**	1.96-3.28	3.38±1.71 n = 37	3.63± 1.48 n = 33	3.88±1.01 n = 11	3.49± 1.61 n = 66	3.72± 1.52 n = 63	3.93± 2.02 n = 46	4.78±1.69 n = 12	3.81±1.74 n = 110
**HDLC**	1.04-1.55	0.58± 0.26 n = 37	0.63± 0.24 n = 29	0.65 ±0.28 n = 11	0.60± 0.25 n = 66	0.59± 0.21 n = 63	0.65± 0.28 n = 46	0.52 ± 0.20 n = 12	0.61± 0,24 n = 110
**RPI**	<2.1	7.35± 5.11 n = 37	6.56± 3.88 n = 29	6.85±3.02 n = 11	7.00± 4.59 n = 66	7.06± 3.63 n = 63	7.37± 7.58 n = 46	12.03± 1.26 n = 12	7.19± 5.62 n = 110
**BMI**	< 25	22.24± 2.27 n = 38	29.69± 6.25 n = 32	35.24± 7.35 n = 12	25.64± 5.86 n = 70	21.67± 2.28 n = 71	29.13± 3.78 n = 12	34.36±3.46 n = 12	24.75± 4.74 n = 121

P values: P values: Age = 0.699, TG = 0.193, TC = 0.232, LDLC = 0.724, HDLC. = 0.815, RPI = 0.080, BMI = 0.251. (Measures of association: age= 0.018, TG= 0.001, TC= 0.015, LDLC= 0.010, HDLC= 0.001, BMI= 0.007). TG= Triglycerides, TC= Total cholesterol, LDL= Low density lipoprotein, HDL= High density lipoprotein, RPI= Risk predictive index, BMI= Body mass index

**Table 2:** Prevalence of dyslipidaemia in relation to alcohol consumption and BMI

	Alcohol Drinkers				Non-alcohol Drinkers			
	Those with Normal BMI (A)	All those with Higher than normal BMI B)	Only those Overtly Obese (C)	All Alcohol drinkers (D)	Normal BMI (E)	Higher than normal BMI (F)	Overtly Obese (G)	All Non-alcohol drinkers (H)
High TG	24.3% n = 8	27.6% n = 8	36.4% n = 4	25.8% n = 17	12.7% n = 8	26.1% n = 12	16.7% n = 2	18.0% n = 20
High TC	27.0% n = 10	31.0% n = 9	36.4% n = 4	28.8% n = 19	31.7% n = 20	45.7% n = 21	58.3% n = 7	37.3% n = 41
High LDLC	51.4% n = 19	58.6% n = 17	72.3% n = 8	54.5% n = 36	55.6% n = 35	63.0% n = 29	83.3% n = 10	59.1% n = 65
Low HDL	97.3% n = 1	93.1% n = 27	90.9% n = 10	95.5% n = 63	95.2% n = 27	93.5% n = 43	100% n = 0	94.5% n = 103

P values: Alcohol drinkers: (High TG: 0.491, High TC: 0.463, High LDL C: 0.368, Low HDLD: 0.408); Non-alcohol drinkers: (High TG: 0. 0.063, High TC: 0.100, High LDL C: 0.279, Low HDLC: 0.064); Raised TG, TC or LDLC = hyperlipidemias; High TC/LDLC and/ or High TG + low HDLC level = combined dyslipidemia

## Discussion

This study found lower total cholesterol (TC) value (4.54mmol/L) than previous but recent studies in different communities in Nigeria [5–8]. The differences in the setting, nutrition and possibly alcohol intake between this and those other communities may account for the differing values, as diet is known to influence TC and other lipids [9]. Several studies have documented higher TC values in heavy eaters and heavier alcohol drinkers [5, 10]. Compared to some other studies in Nigeria and elsewhere, this study found higher mean LDLC and Tg with lower HDLC values probably because those studies involved more educated Nigerians who possibly had better knowledge of healthier diet/ lifestyle that affected lipids favourably. Whereas the TC value fell within normal range, LDLC (atherogenic) was higher and HDLC (antiatherogenic) lower than acceptable desirable values. Other studies [6–8, 11] have also demonstrated low HDLC values in Nigerians. It is possible that among other things, low exercise level may be a major factor in low HDLC levels in Nigerians as studies [12] have shown that as much as 25 to 57% of Nigerians were physically inactive. Thus, measures to lower LDLC and others to increase HDLC should be encouraged in Nigeria and Africa as a whole. Suburban areas need particular attention as they harbor majority of people in the cities in Africa [4].

In agreement with previous studies [9, 13], the mean TC and LDLC in this study increased with BMI irrespective of alcohol consumption status. However, mean TG and HDLC increased linearly with BMI in alcohol drinkers but in non alcohol drinkers, it increased with their BMI and then dropped in those overtly obese. Similarly, except for hypertriglyceridemia prevalence which dropped in overtly obese non alcohol drinkers, the prevalence of all the hyperlipidemias increased with BMI in both groups. These finding conform to some other studies [14] which found BMI as an independent predictor of variation in plasma TG after alcohol consumption. It also indicates/ suggests stronger influence of alcohol on TG more than on cholesterol. The increase in cholesterol values with BMI and the finding of higher mean HDLC value in those with higher than normal BMI (0.64mmol/L) compared to those who were obese (0.58mmol/L) in this study conforms to the findings of other studies [9] which found hypercholesterolemia and HDLC values respectively having positive and inverse associations with Body Mass Index (BMI). Higher HDLC value in obese alcohol drinkers (0.65mmol/L) compared to obese non drinkers (0.52mmol/L) buttresses the HDL raising effect of alcohol [15].

Comparatively, alcohol drinkers generally had lower prevalence of high TC (28.8% vs. 37.3%), high LDLC (54.5% vs. 59.1% and mean risk predictive index (RPI; 7.00 vs. 7.19) than non drinkers; emphasizing the cardiovascular benefit of alcohol consumption. The trend was same whether all those having higher than normal BMI were considered together or only those overtly obese considered separately. For those with normal BMI, however, non alcohol drinkers had better RPI (7.06) than alcohol drinkers (7.35), thus, suggesting that greater cardiovascular benefit from alcohol consumption may possibly be seen the closer the tendency to obesity. However, not being statistically significant may suggest that alcohol consumption alone may not be enough to give the desired level of the protective HDLC and so, further studies are needed to explore or otherwise refute this possibility.

The percentage of those with high (favorable) HDL level was highest in daily alcohol consumers (8.6%) followed by non drinkers (5.6%) and then none of weekly drinkers. This tends to support researchers [15] who advocated that despite beneficial cardiovascular effect of moderate alcohol consumption in most populations; clinical advice for abstainers to initiate daily alcohol consumption should be considered with caution on individual basis. Therefore, other factors which improve HDLC like regular exercise and increased vegetable intake should be encouraged even in those who consume alcohol.

Mean Lipid parameters in this study as well as mean BMI and age were significantly associated with alcohol consumption. Non-alcohol drinkers had lower BMI (24.75Kg/M2) than those who drank alcohol (25.64Kg/M2) and this may not be surprising since drinkers at bar shops in a country like Nigeria often indulge in excessive eating of especially different types of meat prepared in different forms at such places. Alcohol drinkers comparatively had higher BMI values across all the BMI categories, thus, buttressing this eating habit in mild to moderate “social” drinkers unlike in heavy alcoholics who have been shown to have reduced lean body mass due to poor nutrition [10]. Previous studies in Nigerians [5] equally found that “social” alcohol drinkers had higher BMI than non drinkers.

The prevalence of combined dyslipidemia in this study (general; 60.2%, in the obese; 73.9%, those with BMI = 25; 62.7%, those with normal BMI; 58.0%) is lower than the finding in one study [7] in South East, Nigeria, although that study involved elderly folks. Besides lower hypertriglyceridemia prevalence, other lipid derangements were higher in these apparently healthy subjects of this study than the prevalence demonstrated recently in hospital patients like diabetics [8]. This is quite worrisome and deserves attention.

Compared with some other studies in different regions of Nigeria [6, 7, 11] and some other African [3] and non African Nations [13], this study generally found higher prevalence of hypertriglyceridemia (21.0%), hypercholesterolemia (34.1%0, high LDLC (57.4%) and combined dyslipidemias (60.2%) than documented in those studies. Aside differing settings [3] with differing diets and lifestyle, some of those other studies [3, 13] used TC =6.5mmol/L as cut off for hypercholesterolaemia and involved wider populations and older people [3, 13] all of which influence cholesterol values. Although some of the Nigerian studies were conducted in communities with some dietary differences with this one, this may well mean a rising prevalence since there is no overt difference in the diet of the communities in South East and the part of South- South Nigeria in which the other studies [6, 7, 11] were conducted.

The fact that the prevalence of hypercholesterolemia in these “not highly educated” low income earners in this study was higher than that found recently in professionals (those highly educated with higher earnings and as such higher purchasing power) in Southern Nigeria [6], is striking since their mean age was close to that of participants in this study. Higher cholesterol values have previously been documented in “not well” educated business men compared to better educated government workers [5]. This higher prevalence finding in both rich traders with low education5 and low income non traders with low education (as in this study) than in the highly educated higher income earners [6] suggests that ignorance may be playing a greater role than ascribed to it in the emergence of non communicable disease in Africans and Nigerians in particular.

## Conclusion

Ignorance/ poor nutritional and health education may be key factors in the emergence of non communicable diseases in Africans.
